# Single-cell profiling reveals Müller glia coordinate retinal intercellular communication during light/dark adaptation via thyroid hormone signaling

**DOI:** 10.1093/procel/pwad007

**Published:** 2023-02-21

**Authors:** Min Wei, Yanping Sun, Shouzhen Li, Yunuo Chen, Longfei Li, Minghao Fang, Ronghua Shi, Dali Tong, Jutao Chen, Yuqian Ma, Kun Qu, Mei Zhang, Tian Xue

**Affiliations:** Division of Life Sciences and Medicine, Department of Ophthalmology, The First Affiliated Hospital of USTC, University of Science and Technology of China, Hefei 230026, China; Hefei National Research Center for Physical Sciences at the Microscale, Neurodegenerative Disorder Research Center, CAS Key Laboratory of Brain Function and Disease, University of Science and Technology of China, Hefei 230026, China; Division of Life Sciences and Medicine, School of Life Sciences, University of Science and Technology of China, Hefei 230026, China; Division of Life Sciences and Medicine, Department of Ophthalmology, The First Affiliated Hospital of USTC, University of Science and Technology of China, Hefei 230026, China; Hefei National Research Center for Physical Sciences at the Microscale, Neurodegenerative Disorder Research Center, CAS Key Laboratory of Brain Function and Disease, University of Science and Technology of China, Hefei 230026, China; Division of Life Sciences and Medicine, School of Life Sciences, University of Science and Technology of China, Hefei 230026, China; Division of Life Sciences and Medicine, Department of Ophthalmology, The First Affiliated Hospital of USTC, University of Science and Technology of China, Hefei 230026, China; Hefei National Research Center for Physical Sciences at the Microscale, Neurodegenerative Disorder Research Center, CAS Key Laboratory of Brain Function and Disease, University of Science and Technology of China, Hefei 230026, China; Division of Life Sciences and Medicine, School of Life Sciences, University of Science and Technology of China, Hefei 230026, China; Division of Life Sciences and Medicine, Department of Ophthalmology, The First Affiliated Hospital of USTC, University of Science and Technology of China, Hefei 230026, China; Hefei National Research Center for Physical Sciences at the Microscale, Neurodegenerative Disorder Research Center, CAS Key Laboratory of Brain Function and Disease, University of Science and Technology of China, Hefei 230026, China; Division of Life Sciences and Medicine, School of Life Sciences, University of Science and Technology of China, Hefei 230026, China; Division of Life Sciences and Medicine, Department of Ophthalmology, The First Affiliated Hospital of USTC, University of Science and Technology of China, Hefei 230026, China; Hefei National Research Center for Physical Sciences at the Microscale, Neurodegenerative Disorder Research Center, CAS Key Laboratory of Brain Function and Disease, University of Science and Technology of China, Hefei 230026, China; Division of Life Sciences and Medicine, School of Life Sciences, University of Science and Technology of China, Hefei 230026, China; Division of Life Sciences and Medicine, Department of Ophthalmology, The First Affiliated Hospital of USTC, University of Science and Technology of China, Hefei 230026, China; Hefei National Research Center for Physical Sciences at the Microscale, Neurodegenerative Disorder Research Center, CAS Key Laboratory of Brain Function and Disease, University of Science and Technology of China, Hefei 230026, China; Division of Life Sciences and Medicine, School of Life Sciences, University of Science and Technology of China, Hefei 230026, China; Division of Life Sciences and Medicine, School of Life Sciences, University of Science and Technology of China, Hefei 230026, China; Division of Life Sciences and Medicine, Department of Ophthalmology, The First Affiliated Hospital of USTC, University of Science and Technology of China, Hefei 230026, China; Hefei National Research Center for Physical Sciences at the Microscale, Neurodegenerative Disorder Research Center, CAS Key Laboratory of Brain Function and Disease, University of Science and Technology of China, Hefei 230026, China; Division of Life Sciences and Medicine, School of Life Sciences, University of Science and Technology of China, Hefei 230026, China; Division of Life Sciences and Medicine, Department of Ophthalmology, The First Affiliated Hospital of USTC, University of Science and Technology of China, Hefei 230026, China; Hefei National Research Center for Physical Sciences at the Microscale, Neurodegenerative Disorder Research Center, CAS Key Laboratory of Brain Function and Disease, University of Science and Technology of China, Hefei 230026, China; Division of Life Sciences and Medicine, School of Life Sciences, University of Science and Technology of China, Hefei 230026, China; Division of Life Sciences and Medicine, Department of Ophthalmology, The First Affiliated Hospital of USTC, University of Science and Technology of China, Hefei 230026, China; Hefei National Research Center for Physical Sciences at the Microscale, Neurodegenerative Disorder Research Center, CAS Key Laboratory of Brain Function and Disease, University of Science and Technology of China, Hefei 230026, China; Division of Life Sciences and Medicine, School of Life Sciences, University of Science and Technology of China, Hefei 230026, China; Division of Life Sciences and Medicine, Department of Ophthalmology, The First Affiliated Hospital of USTC, University of Science and Technology of China, Hefei 230026, China; Hefei National Research Center for Physical Sciences at the Microscale, Neurodegenerative Disorder Research Center, CAS Key Laboratory of Brain Function and Disease, University of Science and Technology of China, Hefei 230026, China; Division of Life Sciences and Medicine, School of Life Sciences, University of Science and Technology of China, Hefei 230026, China; Division of Life Sciences and Medicine, Department of Ophthalmology, The First Affiliated Hospital of USTC, University of Science and Technology of China, Hefei 230026, China; Hefei National Research Center for Physical Sciences at the Microscale, Neurodegenerative Disorder Research Center, CAS Key Laboratory of Brain Function and Disease, University of Science and Technology of China, Hefei 230026, China; Division of Life Sciences and Medicine, School of Life Sciences, University of Science and Technology of China, Hefei 230026, China; Division of Life Sciences and Medicine, Department of Ophthalmology, The First Affiliated Hospital of USTC, University of Science and Technology of China, Hefei 230026, China; Hefei National Research Center for Physical Sciences at the Microscale, Neurodegenerative Disorder Research Center, CAS Key Laboratory of Brain Function and Disease, University of Science and Technology of China, Hefei 230026, China; Division of Life Sciences and Medicine, School of Life Sciences, University of Science and Technology of China, Hefei 230026, China; Center for Excellence in Brain Science and Intelligence Technology, Chinese Academy of Sciences, Shanghai 200031, China; Institute for Stem Cell and Regeneration, Chinese Academy of Sciences, Beijing 100864, China

**Keywords:** single cell, Müller glial cells, intercellular communication, light/dark adaptation, thyroid hormone signaling

## Abstract

Light adaptation enables the vertebrate visual system to operate over a wide range of ambient illumination. Regulation of phototransduction in photoreceptors is considered a major mechanism underlying light adaptation. However, various types of neurons and glial cells exist in the retina, and whether and how all retinal cells interact to adapt to light/dark conditions at the cellular and molecular levels requires systematic investigation. Therefore, we utilized single-cell RNA sequencing to dissect retinal cell-type-specific transcriptomes during light/dark adaptation in mice. The results demonstrated that, in addition to photoreceptors, other retinal cell types also showed dynamic molecular changes and specifically enriched signaling pathways under light/dark adaptation. Importantly, Müller glial cells (MGs) were identified as hub cells for intercellular interactions, displaying complex cell‒cell communication with other retinal cells. Furthermore, light increased the transcription of the deiodinase *Dio2* in MGs, which converted thyroxine (T4) to active triiodothyronine (T3). Subsequently, light increased T3 levels and regulated mitochondrial respiration in retinal cells in response to light conditions. As cones specifically express the thyroid hormone receptor *Thrb*, they responded to the increase in T3 by adjusting light responsiveness. Loss of the expression of *Dio2* specifically in MGs decreased the light responsive ability of cones. These results suggest that retinal cells display global transcriptional changes under light/dark adaptation and that MGs coordinate intercellular communication during light/dark adaptation via thyroid hormone signaling.

## Introduction

The vertebrate retina is a specialized light-sensitive tissue that consists of various types of neurons and glial cells. Photoreceptors as well as bipolar cells (BCs), amacrine cells (ACs), horizontal cells (HCs), ganglion cells (GCs), and glial cells are organized in an elaborate network that converts light into electrical signals, which are then relayed to the brain for visual perception (Yi et al., 2021). Rod photoreceptors are sensitive to darkness and are thus responsible for scotopic (dark) vision, while cone photoreceptors are adapted to a broad range of light intensities and are responsible for photopic (light) vision. As a fundamental physiological process, light adaptation plays an important role in visual information processing, enabling vertebrates to distinguish light intensities from starlight to sunlight (Fain et al., 2001; Luo et al., 2008; Morshedian and Fain, 2017). Light-adapted photoreceptors intrinsically reduce their light sensitivity and accelerate light response kinetics, allowing the retina to adapt to background illumination rapidly (Fu and Yau, 2007; Morshedian and Fain, 2017).

The mechanisms underlying light adaptation rely on phototransduction in photoreceptors, synaptic transmission, and neuromodulation. Calcium-dependent phototransduction is considered a primary mechanism of light adaptation in vertebrate photoreceptors (Fu and Yau, 2007; Yau and Hardie, 2009). Calcium-binding proteins, which are activated by light-induced low intracellular calcium concentrations, regulate key phototransduction components in a negative feedback manner, enabling photoreceptors to respond continuously to light stimulation under different light backgrounds (Yau, 1991; Fain, 2011; Weiss et al., 2012). In addition, light drives the redistribution of key phototransduction proteins, such as transducin and arrestin, resulting in reduced photoreceptor sensitivity (Zhang et al., 2003; Fu and Yau, 2007). Furthermore, photoreceptors, BCs, HCs, and GCs interact in light/dark adaptation through synaptic transmission. For example, the feedback from HCs onto cones is inhibitory and depolarizes cones under light adaptation (Wen and Thoreson, 2019). GCs receive synaptic transmissions from ACs and BCs to adapt to changes under light by adjusting their sensitivity and temporal filtering characteristics (Liu and Gollisch, 2015). Neuromodulators also play physiological roles in the retina by mediating information processing (Thoreson and Witkovsky, 1999; Witkovsky, 2004). For instance, the neuromodulator dopamine is released by ACs and modulates rod–cone coupling, leading to an increase in cone circuits during light adaptation (Li et al., 2013; Chaffiol et al., 2017). This evidence suggests that retinal cells assemble as a complex network to adapt to dynamic light/dark conditions.

To understand transcriptional changes and cell‒cell communication among various retinal cell types under light/dark adaptation, we performed single-cell RNA sequencing (scRNA-seq) to map 25,176 cell transcriptomes and identified differential single-cell transcriptomes under light/dark adaptation. Based on differentially expressed genes (DEGs) and cell‒cell communication analysis, the results showed that Müller glial cells (MGs) exhibited complex interactions with other retinal cells, and the interaction strength between MGs and other retinal cells was higher in light adaptation, suggesting that MGs play a crucial role in cell‒cell communication during light adaptation. Our results further indicated that thyroid hormone (TH) signaling played a crucial role in coordinating MGs and other retinal cells, especially cones, to modulate light adaptation. Notably, light increased *Dio2* (type 2 deiodinase) transcription in the MGs, thereby increasing triiodothyronine (T3) levels in the retina to regulate mitochondrial respiration in retinal cells, especially in cones with high expression of the thyroid receptor *Thrb*, to adjust to light adaptation. To confirm that *Dio2* in the MG is crucial for cone photosensitivity, we specifically deleted *Dio2* in MGs by injection of AAV-GFAP-Cre virus into *Dio2*^*f*/*f*^ mice. The results indicated that specific loss of *Dio2* in MGs impaired the photoresponse of cones. Thus, our study showed that retinal cells exhibit global transcriptional changes under light/dark adaptation, and MGs coordinate intercellular communication during light/dark adaptation via TH signaling.

## Results

### Landscape of cell‒cell communication under light/dark adaptation through single-cell transcriptomes

**Figure 1.**
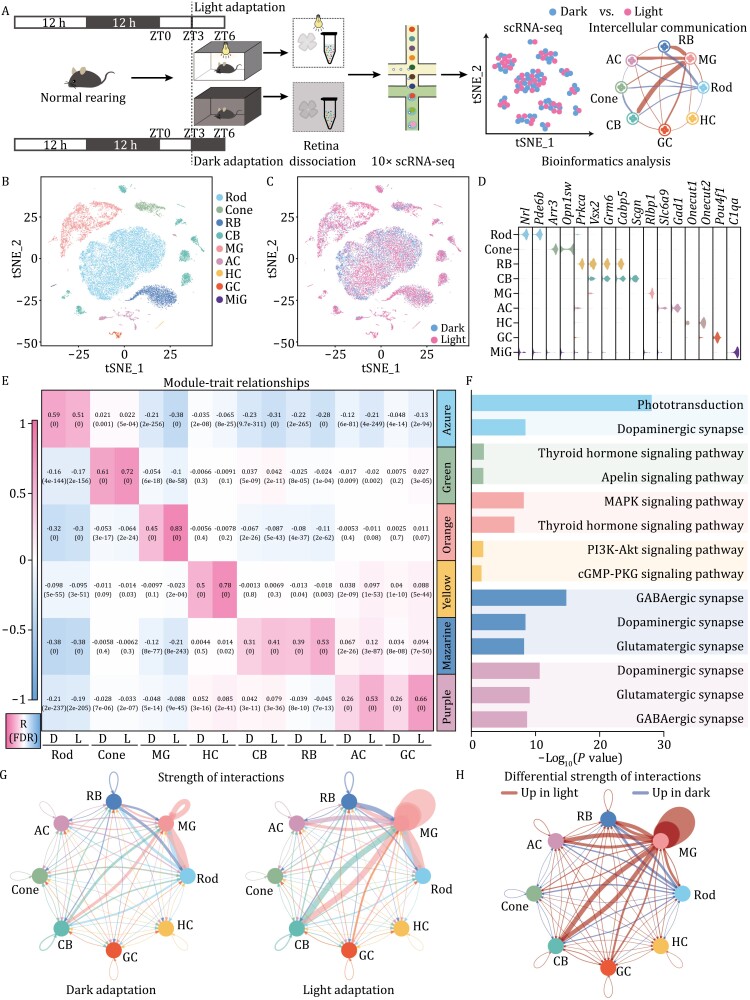
**Landscape of cell‒cell communication and pathways under light/dark adaptation through single-cell transcriptomics.** (A) Experimental workflow of single-cell sample preparation and data analysis under light/dark adaptation in mice. (B and C) t-SNE plot of 25,176 cells from different adaptation samples. Each cell type was defined by cell-type specific marker genes. Cell clusters were color-coded and annotated based on transcriptional profile identities (RB, rod bipolar; CB, cone bipolar; MG, Müller glia; AC, amacrine cell; HC, horizontal cell; GC, ganglion cell; MiG, microglia). (D) Violin-plot distributions of the expression levels of the known cell-type-enriched markers in Fig. S1D and S1E. (E) Module–trait relationships. A heatmap of Pearson correlation coefficients between major retinal cell types and module Eigengenes was defined by WGCNA under light/dark adaptation. (F) KEGG pathways enriched in modules of different cell types. (G) Circos plot of interactions between each cell type under different adaptation conditions. The width of junction lines between different groups of cells was proportional to the strength of ligand‒receptor interactions between groups of cells. (H) Circos plot of the differential strength of interactions between each cell type under different adaptation conditions. Red and blue lines show enhanced cell connections under light adaptation and dark adaptation, respectively.

Molecular characterization of dark- and light-adapted mouse retinae at single-cell resolution was analyzed using droplet-based scRNA-seq on the 10× Genomics platform (Fig. 1A). In total, we established the single-cell transcriptomic profiles of 25,176 retinal cells, including 14,909 cells from light-adapted retinae and 10,267 cells from dark-adapted retinae. Sequencing depth and quality were analyzed by the count number, gene number per cell, and molecules per cell in each sample (Fig. S1A–C and Table 1). We then performed unbiased clustering of the single-cell profiles and identified 17 clusters. Using known retinal cell-type markers, we grouped the 17 clusters into nine major types, including seven neuronal cell types, i.e., rods, cones, rod bipolar cells (RBs), cone bipolar cells (CBs), HCs, ACs, and GCs, and two glial cell types, i.e., MGs and microglia (MiG) (Figs. 1B–D, S1D and S1E). Therefore, all major retinal cell types were identified in our samples based on scRNA-seq analysis.

**Table 1.** Quality analysis of different sequencing data.

Sample ID	Number of cells	Mean reads/cell	Genome mapping (%)	Exon mapping (%)	Intronic mapping (%)	Intergenic mapping (%)
1: Quality analysis of single-cell sequencing data from retina under dark/light adaptation						
retina_1(Dark)	4,923	105,806	95.2	61.1	26.8	5.3
retina_2(Light)	5,152	81,694	95.1	58.4	29.0	5.8
retina_3(Light)	3,618	56,175	93.8	57.0	29.5	5.5
retina_4(Dark)	7,009	140,602	95.1	65.9	21.4	5.8
retina_5(Light)	8,149	119,872	95.7	65.0	23.2	5.5
2: Quality analysis of single-cell sequencing data from retina *ex vivo* incubation						
T3_1	4,880	97,415	94.6	60.7	26.4	5.6
T3_2	5,941	76,591	94.2	59.1	27.9	5.5
Control	4,562	102,664	95.6	62.7	25.7	5.4

Sample ID	Total raw reads		Overall alignment rate (%)		Final mapped reads	
3: Quality analysis of bulk RNA sequencing data from retina under dark/light adaptation						
retina-light_1	34,010,142		92.86		31,583,075	
retina-light_2	33,979,178		92.88		31,559,378	
retina-dark_1	33,899,728		92.75		31,440,549	
retina-dark_2	33,371,183		92.45		30,850,759	

To investigate the molecular changes in cells under light/dark adaptation, we performed weighted gene correlation network analysis (WGCNA) to identify cell-type-specific correlated modules. The enriched pathways in the modules were then analyzed by Kyoto Encyclopedia of Genes and Genomes (KEGG) pathway analysis (Fig. 1E and 1F). We identified six colored modules correlated with characteristic cell types under light/dark adaptation, including the rod-associated azure module and cone-associated green module. Interestingly, MG correlations showed the strongest changes under light/dark conditions. Pathway analysis indicated that each module had its own specific enriched pathway. For instance, the dopamine signaling pathway was enriched in rod-, BC-, AC-, and GC-related modules, matching its function in regulating neural circuits and circadian rhythm (Goel and Mangel, 2021). As two common neurotransmitters in the retina, GABAergic and glutamatergic synapses are enriched in various interneurons involved in the dynamic regulation of light/dark adaptation (Ralph and Menaker, 1989; Della Maggiore and Ralph, 1999; Uckermann et al., 2004; Herrmann et al., 2011; Chaffiol et al., 2017). We found that the TH signaling pathway was enriched in both cone- and MG-related modules (Fig. 1F), suggesting that MGs and cones may cooperate under light adaptation via TH signaling. Taken together, single-cell profiling revealed enriched gene sets and signaling pathways in each retinal cell under light/dark adaptation.

To explore whether retinal cells communicate with each other via molecular interactions under light/dark adaptation, we constructed a cell‒cell communication network based on retinal cell type using CellChat, which is widely applied for the exploration of cell‒cell communication with scRNA-seq data. The results showed complex cell communication, with stronger interactions under light than dark conditions (Fig. 1G). Notably, MGs showed stronger interactions with other retinal cells, especially under light conditions (Fig. 1H). These results imply that MGs may work as a key hub in cell‒cell communication during light/dark adaptation.

### Differential single-cell transcriptomes of mouse retinae under light/dark adaptation

**Figure 2.**
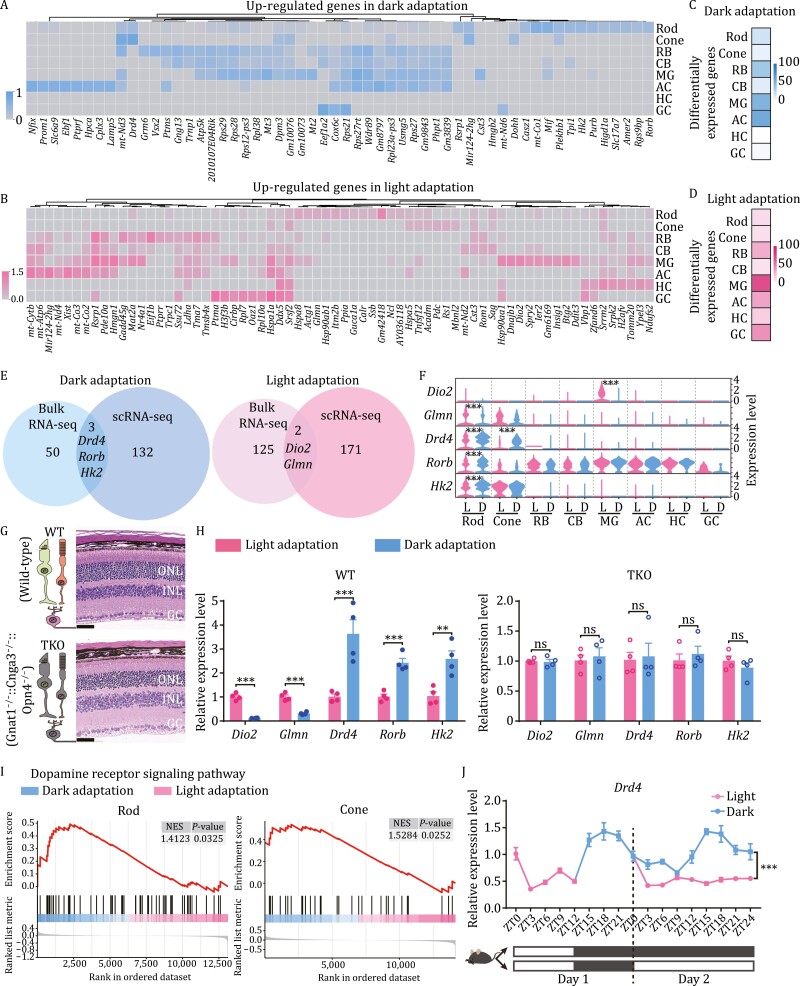
**Differential single-cell transcriptomes of mouse retinae under light/dark adaptation.** (A and B) Heatmap of representative DEGs in retinal cells under light/dark adaptation. (C and D) Quantification of DEG number in each cell type under light/dark adaptation. (C) Number of upregulated genes under dark adaptation. (D) Number of upregulated genes under light adaptation. Filter conditions are described in “Methods”. (E) Venn diagrams of DEGs overlapping between bulk RNA-seq and scRNA-seq under light/dark adaptation. (F) Violin plots showing the expression distribution of DEGs in Fig. 2E in each retinal cell type under light and dark adaptation. ^***^P < 0.001. (G) Hematoxylin and eosin (HE) staining of retinal cross-sections (ONL, outer nuclear layer; INL, inner nuclear layer; GC, ganglion cell) on flat-mounted retinas showing no difference in retinal morphology between WT and TKO mice. Scale bar, 50 μm in HE−stained images. (WT, wild-type mouse; TKO, *Gnat1*^−/−^, *Cnga3*^−/−^, *Opn4*^−/−^ mouse). (H) Comparison of whole-retinal RNA samples confirmed that the expression of DEGs under light/dark adaptation was related to retinal photosensitivity. Each sample contained two retinae, *n* = 4; ^**^P < 0.01; ^***^P < 0.001. (I) GSEA enrichment diagram of changes in dopamine receptor signaling pathways in rods and cones under light/dark adaptation. (J) During a 48-h adaptation experiment, detection and comparison of retinal RNA samples showed that changes in *Drd4* gene expression during light/dark adaptation were mainly influenced by inhibition of light compared with changes in endogenous circadian rhythm. Each sample contained two retinae. *n* = 4; ^***^P < 0.001, Two-way ANOVA.

To investigate the differential molecular features of retinal cells under light/dark adaptation, we analyzed the DEGs in each retinal cell cluster. The results showed that 135 genes were upregulated in dark adaptation and 173 genes were upregulated in light adaptation (Fig. 2A and 2B). All DEGs are listed in Table S1. As expected, several known genes related to visual adaptation were upregulated, including the key phototransduction arrestin gene *Sag* and the GCAP1 coding gene *Guca1a* (Codega et al., 2009). Moreover, we found that *Drd4* was highly expressed in dark-adapted photoreceptors and that *Dio2* was highly expressed in light-adapted MGs. Dopamine is released by dopaminergic ACs and diffuses in the retina to act on receptors in different cells (Witkovsky, 2004). *Dio2* is a deiodinase present in glial cells that converts T4 to T3 and acts on surrounding neurons through the expression of corresponding receptors (Eldred et al., 2018). These data further supported that cell‒cell communication was involved in photoreceptor adaptation. Interestingly, heat shock genes, including *Hsp90aa1*, *Hsp90ab1*, *Hspa5*, and *Hspa8,* were also highly expressed in the light-adapted retina. These proteins can mediate lysosomes to refold unfolded proteins, thus protecting cells from the effects of protein toxicity (Outeiro et al., 2006; Park et al., 2007). Thus, light likely causes stress in retinal cells and triggers the expression of heat shock proteins to protect photoreceptors. We also counted the number of enriched DEGs in each cell type and found that all retinal cells showed variable changes in transcriptomes in response to light/dark adaptation. Furthermore, MGs were the most responsive cell type in both light/dark adaptation (Fig. 2C and 2D).

We also integrated the bulk RNA-seq and scRNA-seq data, with the related DEGs listed in Table S2. *Drd4*, *Rorb*, and *Hk2* were highly expressed in the dark-adapted retinae, and *Glmn* and *Dio2* were highly expressed in light-adapted retinae (Fig. 2E). Notably, *Drd4* showed significant differences in gene expression between light and dark adaptation in the photoreceptors, and *Dio2* was specifically and highly expressed in the MGs under light conditions (Fig. 2F). To validate the differential expression of these genes under light/dark adaptation, we performed quantitative real-time polymerase chain reaction (qRT-PCR) in wild-type and *Gnat1*^−/−^; *Cnga3*^−/−^; *Opn4*^−/−^ triple knockout mice (TKO) (Fig. 2G and 2H). *Gnat1* encodes a phototransduction protein in rods, *Cnga3* encodes a cyclic nucleotide-gated channel subunit in cones, and *Opn4* encodes the light-sensitive protein melanopsin in intrinsically photosensitive retinal GCs (ipRGCs). Thus, TKO mice lack essential components of the phototransduction pathway in rods, cones, and ipRGCs and therefore lack all sensitivity to light (Hattar et al., 2002; Fu and Yau, 2007). Our results showed that *Glmn*, *Dio2*, *Drd4*, *Rorb*, and *Hk2* were differentially expressed in the light- and dark-adapted wild-type retinae but not in the TKO mice (Fig. 2H), indicating that the expression of these DEGs was light dependent.

Dopamine is the most studied neuromodulator under light/dark adaptation. Thus, we further investigated the dopamine receptor signaling pathway using gene set enrichment analysis (GSEA). The results confirmed that the dopamine receptor signaling pathway was enriched in both dark-adapted rods and cones (Fig. 2I). Circadian changes in *Drd4* mRNA levels have been observed in previous studies (Jackson et al., 2011). Therefore, to clarify whether *Drd4* expression is primarily regulated by light rather than circadian rhythm, we monitored *Drd4* expression for 48 h. The results indicated that *Drd4* expression changed with endogenous circadian rhythm fluctuations [day 1: zeitgeber time (ZT) 0–ZT24 under normal rearing conditions; day 2: ZT0–ZT24 under dark condition] (Fig. 2J) but was significantly reduced under light condition (day 2: ZT12–ZT24). Therefore, changes in *Drd4* expression during adaptation are largely light inhibited, although *Drd4* also shows its own circadian pattern. Taken together, these findings are consistent with the scRNA-seq results and strongly suggest that DEG gene expression is regulated by light.

### Light activates the expression of MG-specific *Dio2*

**Figure 3.**
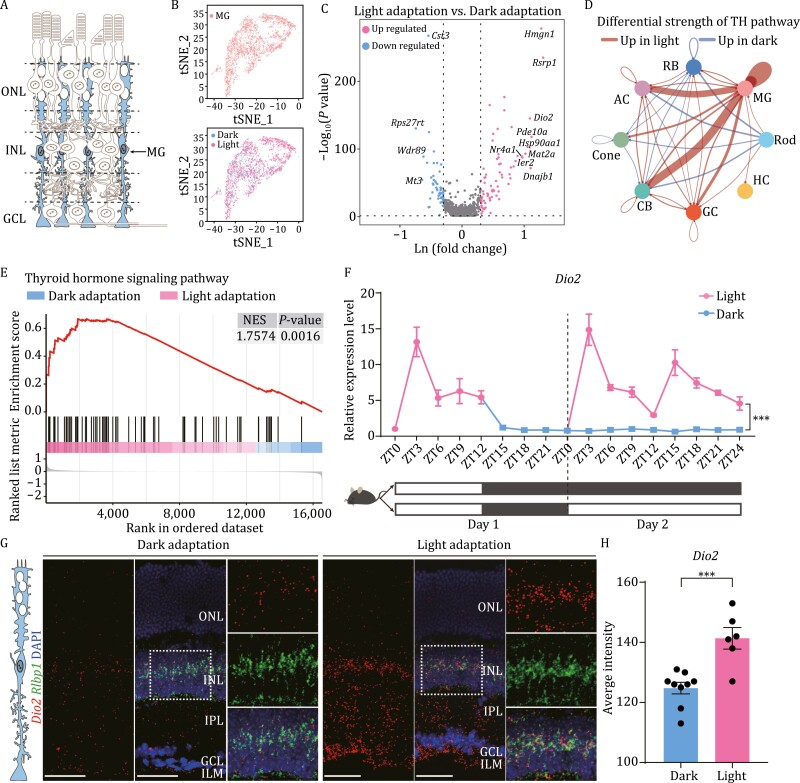
**The *Dio2* expression in retinal MG cells under light adaptation.** (A) Schematic of the morphology and localization of MGs in mouse retina. (B) t-SNE plot of 4,246 MG cells under light/dark adaptation, 988 from dark-adapted samples and 3,258 from light-adapted samples. (C) Volcano plot of DEGs in mouse MGs under light/dark adaptation. Filter conditions are described in “Methods” as in Fig. 2A and 2B. (D) Circos plot of the differential strength of interactions in thyroid hormone (TH) pathways between each cell type under different adaptation conditions. Red and blue lines show enhanced cell connections under light adaptation and dark adaptation, respectively. (E) GSEA enrichment diagram of the TH pathway in MGs under light/dark adaptation. (F) The expression of *Dio2* was activated by light during light/dark adaptation. Each sample contained two retinae. *n* = 4; ^***^P < 0.001, Two-way ANOVA. (G) Representative images of dual RNAscope *in situ* hybridization of *Dio2* (labeled in red) and *Rlbp1* (labeled in green) under light/dark adaptation in mouse retinae. Blue, DAPI (nuclear marker). Scale bar, 50 μm. Experiments were repeated three times independently with similar results. (IPL, inner plexiform layer; ILM, inner limiting membrane). (H) Quantification of *Dio2* intensity signals per unit area under different adaptation conditions. ^***^P < 0.001. Data are representative of three independent experiments.

As a class of primary glial cells in the retina, MGs provide nutritional and physiological support for other retinal cells as well as 11-*cis* retinol for the cone visual cycle (Wang and Kefalov, 2009; Morshedian et al., 2019) (Fig. 3A). Our study suggests that MGs play a significant role in the process of light adaptation and communicate with other retinal cells via the TH pathway (Fig. 1E–H). Analysis of the DEGs in the MGs showed that *Dio2* expression was higher under light adaptation than under dark adaptation (Fig. 3B and 3C). Furthermore, based on CellChat analysis, the interaction strength of the *Dio2*-related TH pathway was upregulated in MGs under light conditions (Fig. 3D). GSEA also illustrated that the TH signaling pathway was enriched in MGs under light adaptation (Fig. 3E).

We next examined the expression of *Dio2* in adult wild-type mice under different light and dark environments. The results indicated that *Dio2* expression changed with endogenous circadian rhythm fluctuation but was significantly upregulated under light conditions (day 1: ZT0–ZT12 in normal rearing condition; day 2: ZT0–ZT24 in light condition) (Fig. 3F), suggesting that light activates *Dio2* expression in light-adapted retinae. To further verify the changes in *Dio2* mRNA expression with light conditions, RNAscope analysis was performed to verify the location and mRNA expression of *Dio2*. The results showed that *Dio2* signaling was enhanced in the light-adapted retinae, primarily in the inner layer of retinal nuclear cells, inner plexiform layer, and end foot of the inner limiting membrane, the same as the location of MGs (Fig. 3G and 3H). These findings are consistent with the single-cell profiling results showing high and specific expression of *Dio2* in MGs under light conditions (Fig. 2F). These results confirm that *Dio2* expression and the TH pathway are upregulated in MGs under light adaptation.

### Light increases T3 modulation of single-cell transcriptomes of retinal cells

**Figure 4.**
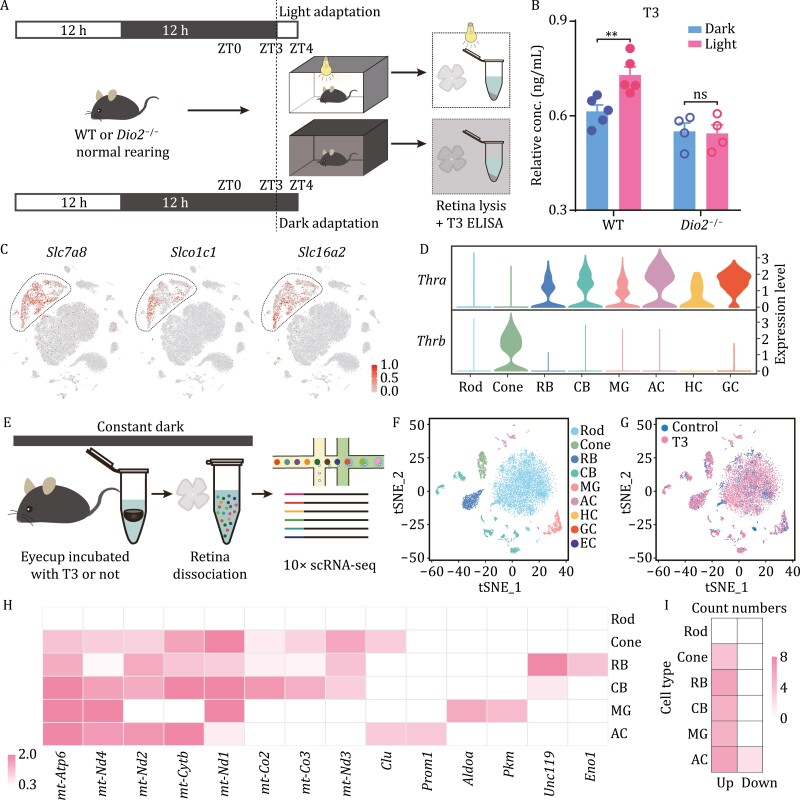
**Participation of MGs in activation of the TH pathway under light adaptation.** (A) Experimental workflow of T3 ELISA sample preparation under light and dark adaptation. (B) ELISA showing that the T3 concentration in mouse retinae increased significantly after 1 h of light adaptation (*n* = 5), but this increase was not observed in *Dio2* knockout mice (*n* = 4). Each sample contained four retinae. ^**^P < 0.01. (C) Visualization of TH transporter expression (*Slc7ab*, *Slco1c1*, and *Slc16a2*) by t-SNE in mouse retinal samples. Cells are colored according to gene expression levels (red, high; gray, low). (D) The expression pattern of the TH receptors, *Thra* and *Thrb,* in mouse retinae. (E) Experimental workflow of single-cell sample preparation under *ex vivo* T3 incubation. (F and G) t-SNE plot of 10,085 cells under light/dark adaptation. A total of 6,000 cells were isolated from T3 incubation, and 4,085 cells were isolated in the control. Each cell type was defined by well-known retinal cell markers. Cell clusters are color coded and annotated based on transcriptional profile identities. (H) Heatmap of the expression changes of some DEGs in different cell types under *ex vivo* T3 incubation (with no significant changes in genes in rods). (I) The number of DEGs in each cell group under different conditions was calculated. Filter conditions are described in “Methods”.

As DIO2 can regulate local T3 levels (Bianco and Kim, 2006), we next explored whether light-activated expression of *Dio2* in MGs can increase T3 levels in the retina (Fig. 4A). The results showed a significant increase in T3 levels in the retina under light condition (Fig. 4B). However, the differences were abrogated in *Dio2* knockout mice. The data suggest that the change in T3 concentration in the retina is largely dependent on *Dio2* activity. TH transporters regulate the cellular influx and efflux of T4 and T3, thus, TH transporters were widely expressed in different retinal cells (Fig. S2A), and *Slc7a8*, *Slco1c1*, and *Slc16a2* were enriched in MGs (Fig. 4C). However, their expression levels were not changed significantly during light/dark adaptation in a cell type-specific manner (Fig. S2B).

Of the two TH receptor subtypes, *Thrb* is specifically expressed in the cones, and *Thra* is broadly expressed in retinal cells other than photoreceptors. Interestingly, we found that TH receptors were not expressed in the rods (Fig. 4D). To investigate the effect of the TH pathway on the transcription of retinal cells, we performed scRNA-seq on mouse retinae incubated with T3 (Figs. 4E–G and S2C–G). All DEGs are listed in Table 2. As *Thrb* and *Thra* were expressed in a cell-type-specific manner, the T3 upregulated genes were primarily detected in cones, BCs, GCs, and MGs with *Thrb* and *Thra* expression. As *Thra* and *Thrb* were not expressed in the rods, no responsive genes were identified in rods compared to other retinal cells. Interestingly, most DEGs were upregulated after T3 incubation, including a set of mitochondrial respiration-related genes (Figs. 4H, 4I and S2H).

**Table 2.** Differentially expressed genes of different cell types with *ex vivo* T3 incubation.

Gene	avg_logFC	pct.1	pct.2	*P*_value_adjust	Cell type
*mt-Nd1*	2.07966	0.655	0.064	1.69 × 10^−124^	Cone_bipolar
*mt-Co2*	1.758067	0.653	0.095	1.76 × 10^−106^	Cone_bipolar
*mt-Co3*	1.414253	0.76	0.217	5.78 × 10^−94^	Cone_bipolar
*mt-Nd4*	1.632519	0.556	0.07	1.80 × 10^−87^	Cone_bipolar
*mt-Atp6*	3.505685	0.334	0	5.04 × 10^−73^	Cone_bipolar
*mt-Nd1*	3.218472	0.759	0.023	2.91 × 10^−81^	Cone
*mt-Nd3*	1.528561	0.507	0	1.40 × 10^−49^	Cone
*mt-Cytb*	1.589707	0.877	0.312	3.66 × 10^−47^	Cone
*mt-Atp6*	1.138516	0.912	0.555	8.86 × 10^−34^	Cone
*mt-Co3*	2.711515	0.794	0.333	0.000305675	Horizontal
*Aldoa*	1.507862	0.361	0	6.05 × 10^−34^	Muller
*mt-Nd4*	2.19117	0.292	0	1.14 × 10^−25^	Muller
*Pkm*	1.226279	0.285	0	7.15 × 10^−25^	Muller
*mt-Atp6*	3.057879	0.271	0	2.72 × 10^−23^	Muller
*mt-Nd1*	2.646601	0.261	0	4.04 × 10^−22^	Muller
*mt-Atp6*	1.449856	0.893	0.403	2.22 × 10^−53^	Rod_bipolar
*Unc119*	1.966066	0.446	0	1.64 × 10^−51^	Rod_bipolar
*mt-Cytb*	1.103454	0.906	0.611	1.06 × 10^−36^	Rod_bipolar
*mt-Nd2*	1.476878	0.565	0.102	2.35 × 10^−36^	Rod_bipolar
*mt-Nd3*	1.130941	0.55	0.132	1.48 × 10^−28^	Rod_bipolar
*Eno1*	1.134283	0.503	0.142	6.52 × 10^−23^	Rod_bipolar
*mt-Atp6*	2.277279	0.531	0.055	2.89 × 10^−12^	Amacrine
*Ssu72*	−1.283021	0.011	0.33	7.34 × 10^−11^	Amacrine
*mt-Nd4*	1.694686	0.5	0.077	6.87 × 10^−10^	Amacrine
*Clu*	1.014919	0.328	0	1.40 × 10^−8^	Amacrine
*mt-Nd2*	1.774477	0.477	0.088	4.20 × 10^−7^	Amacrine
*mt-Cytb*	3.347556	0.294	0	5.18 × 10^−7^	Amacrine
*Rps28*	−1.103864	0.122	0.484	8.01 × 10^−7^	Amacrine
*Prom1*	1.043787	0.286	0.011	7.47 × 10^−5^	Amacrine

### The TH pathway modulates retinal mitochondrial respiration and cone photoresponse

**Figure 5.**
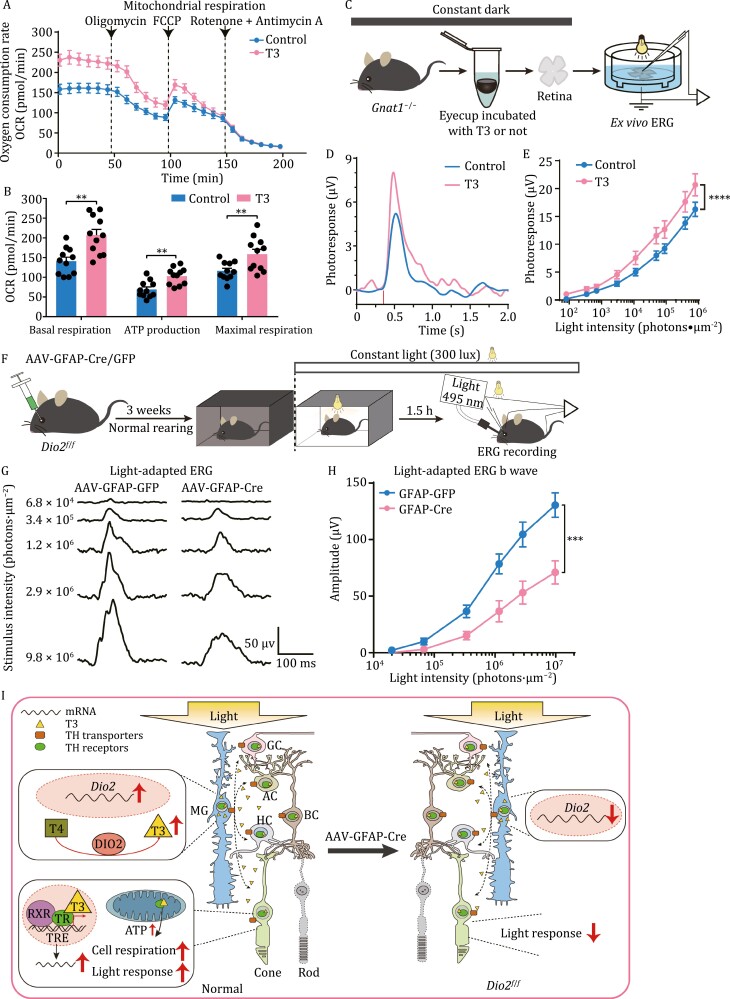
**Activation of TH pathways under light adaptation regulates retinal mitochondrial respiration and cone photoresponse.** (A and B) Seahorse analysis of the oxygen consumption rate (OCR) of the mouse retinae treated with 100 nmol/L T3 incubation *ex vivo*. OCR was measured as baseline measurements and in the presence of the indicated drugs, i.e., 20 μmol/L oligomycin, 1 μmol/L FCCP, and 1 μmol/L rotenone with 1 μmol/L antimycin A (R + A). Data are representative of three independent experiments. *n* = 11, ^**^P < 0.01. (C) Schematic of the *ex vivo* ERG for T3-incubated mouse retinae. *Gnat1*^−/−^ mice lacked transducin α protein, resulting in rods that were not photosensitive and cones that were normally photosensitive. (D) Representative flash response images of a-wave under 1.13 × 10^4^ photons•μm^−2^ light. The T3 incubation group showed a higher photocurrent than the control group. (E) Quantification of ERG a-wave amplitudes of light intensity-response curves from cones in normal incubation and T3 incubation. The light intensity ranged from 80.71 to 7.89 × 10^5^ photons/μm^2^. Intensity-response data are mean ± s.e.m. intensity-response curves. *n* = 18, ^****^P < 0.0001, Two-way ANOVA.(F) Schematic diagram of the ERG recording experiment in AAV-GFAP-Cre/GFP virus-injected *Dio2*^*f*/*f*^ mice under light adaptation. The control group was injected with AAV-GFAP-GFP, and the experimental group was injected with AAV-GFAP-Cre-GFP. ERG recordings were performed 3 weeks after the virus injection. (G) Representative flash response images of *in vivo* ERG b-wave under different light intensities. Light with a 495 nm wavelength was used as the stimulus light, a 20 ms flash stimulus was given, and the light intensities were 6.8 × 10^4^, 3.4 × 10^5^, 1.2 × 10^6^, 2.9 × 10^6^, and 9.8 × 10^6^ photons/μm^2^. (H) Quantification of ERG b-wave amplitudes of light intensity-response curves with light intensities from 2.0 × 10^4^ to 9.8 × 10^6^ photons/μm^2^. Intensity-response data were shown as the mean ± s.e.m. intensity-response curves. *n* = 9, ^***^P < 0.001, Two-way ANOVA. (I) Summary showing that the thyroid hormone signaling pathway activated by *Dio2* under light adaptation affects overall retinal mitochondrial respiration and light response.

Mitochondrial genes were the major DEGs with T3 incubation, so we are consequently interested in mitochondrial function in light adaptation. Mitochondria perform various tasks and metabolic functions, including the regulation and maintenance of Ca^2+^ homeostasis, oxidative energy metabolism, and neuronal excitability (Gunter and Pfeiffer, 1990; Erecinska et al., 2004; Nicholls, 2005; Kann and Kovacs, 2007; Esteras et al., 2017). We next examined the effects of T3 on retinal mitochondrial respiration using Seahorse analysis to measure the oxygen consumption rate (OCR) of retinal explants treated with or without T3 (Figs. 5A and S2I). We found that basal respiration, adenosine triphosphate (ATP) production, and maximum respiration were significantly higher in the T3 group than in the control group after *ex vivo* incubation, suggesting that T3 may activate retinal cell metabolism (Fig. 5B).

Cones require high mitochondrial function for survival and metabolism under light conditions (Giarmarco et al., 2020). As *Thrb* is highly and specifically expressed in cones, we used *Gnat1*^−/−^ mice (rod cells insensitive to light) to monitor the effects of T3 on the ability of cones to respond to light using *ex vivo* electroretinography (ERG) (Cai et al., 2019; Li et al., 2021) (Fig. 5C). Light adaptation is known to increase cone-mediated photopic ERG responses (Peachey et al., 1992; Ekesten et al., 1998; Cameron and Lucas, 2009). Based on comparative analysis, the light response amplitude of the ERG a-wave in *Gnat*^−/−^ mice, representing the photoresponse of cones, increased significantly in the T3 incubation group (Fig. 5D and 5E). Furthermore, there was no change in the retinal light response in rod cells between the T3 and control groups using single rod suction pipette recording (Ma et al., 2019) (Fig. S3A–D). To further confirm that *Dio2* in the MGs is crucial for cone photosensitivity, we specifically deleted *Dio2* in the MGs by injection of AAV-GFAP-Cre virus into *Dio2*^*f*/*f*^ mice (Fig. S3E and S3F). Using *in vivo* ERG, we confirmed that specific loss of *Dio2* in MGs impaired the photoresponse of cones (Fig. 5F–H). In contrast, there was no change in the b-wave in dark-adapted ERG analysis (Fig. S3G–J). Thus, we propose that light activates *Dio2* in MGs to generate additional T3 to act on other cells, thereby increasing mitochondrial respiration and cone photoresponses in the retina via the TH pathway (Fig. 5I).

## Discussion

Previous studies have suggested that the mechanisms of photoreceptor light/dark adaptation mainly rely on phototransduction. To determine whether retinal cells respond to light/dark adaptation by changing their molecular and cellular features, we established a detailed single-cell transcriptome of 25,176 retinal cells under light/dark adaptation. Based on scRNA-seq profiling, continuous light and dark exposure triggered a series of cell-type-specific molecular changes and signaling pathways. Interestingly, the cell‒cell communication map of different retinal cells indicated that MGs were a crucial cell type involved in light adaptation, regulating retinal cell mitochondrial metabolism and light responses of cone photoreceptors by catalyzing T4 to T3 using DIO2. Cones utilize a large amount of ATP in long-term light adaptation, such as daylight, because cones never remain saturated and must continue to export Na^+^ and synaptic Ca^2+^ even in bright illumination. The increase in *Dio2* in MGs helps to convert more T4 to T3 and then increase mitochondrial aspiration of cones to generate enough ATP for cone phototransduction. These results elucidate the intercellular communication among retinal cells and their dynamic molecular features responsible for light adaptation. Thus, this study provides a new route to understand the molecular mechanisms underlying light/dark adaptation.

We found that MGs and photoreceptors coordinate to regulate light/dark adaptation via the TH signaling pathway. In the retina, T3 is mainly derived from MGs, and retinal neurons expressing *Thra* and *Thrb* are the primary targets of T3 action. MGs provide homeostatic and metabolic support for retinal neurons and mediate the transport of transcellular ions and cytokines to provide trophic support. Therefore, MGs are reasonably involved in the regulation of the light/dark ability of retinal neurons via cell‒cell communication. Similar to the retina, *Dio*2 expression is also important in astrocytes and targets adjacent neurons via paracrine signaling to activate neuronal gene expression in the brain (Horn and Heuer, 2010; Hernandez et al., 2021). *Dio2* activity in the brain increases spatially and temporally with T3 concentration to act in critical brain processes such as myelination, neuronal migration, and glial differentiation (Rovet, 2014; Zendedel et al., 2016). Collectively, these observations suggest an important role for glial cells in TH homeostasis in the central nervous system and a close coupling between glial cells and neurons in TH metabolism.

Light induces the transcription of *Dio2*, and then *Dio2* can rapidly amplify T3 levels within a few hours in local tissues in the paracrine manner. Conversely, the activity of endogenous DIO2 in cells is post-translationally regulated by the substrate, which, in turn accelerates the degradation of DIO2 through the ubiquitin-proteasome pathway, resulting in a short half-life of DIO2 (Arrojo et al., 2013; Gereben et al., 2015). Thus, the expression of *Dio2* is dynamically controlled through transcriptional and translational regulation, and the activation and deactivation of *Dio2* modulates the T3 concentration. Thus, *Dio2* is an excellent regulator of retinal light/dark adaptation. As the expression and activation of *Dio2* can be triggered by cAMP, it is possible that light-stimulated l-glutamate or dopamine may activate cAMP to promote *Dio2* expression and activation. However, how light induces Dio2 expression needs further study.

TH signaling plays a central role in cone subtype specification during development. Previous studies on retinal organoids have shown that low TH signaling is early to specify S cones and high TH signaling later in development to produce L/M cones (Eldred et al., 2018). However, the function of TH in the adult retina remains unclear. Here, by single-cell sequencing analysis, we found global transcriptional changes in most retinal cells, except rods, following T3 incubation, as well as mitochondrial metabolism in the adult mouse retina. We hypothesized that MGs may be responsible for light stimulation under increased *Dio2* expression, which induces T3 production in the retina and activates the transcriptional expression and mitochondrial respiration of adjacent retinal cells. Furthermore, T3 induces gene transcription via the nuclear receptors *Thra* and *Thrb*, allowing retinal energy to meet the need under light condition. Furthermore, we specifically deleted the expression of *Dio2* in MGs by injecting AAV-GFAP-Cre in *Dio2*^*f*/*f*^ mice. The ERG results proved that loss of *Dio2* in MGs decreased the photoresponse of cones, consistent with our theory. In conclusion, the TH pathway activated by MG cells under light adaptation affects the cone light response by regulating mitochondrial metabolism in the retina.

However, how T3 induces mitochondrial respiration and biogenesis in retinal cells is unknown. Previous data suggest that T3 induces the expression of mitochondrial transcription factor A (TFAM), a nuclear-encoded protein that binds to mtDNA to regulate its transcription (Falkenberg et al., 2002). Another transcription factor, p43, is a truncated form of nuclear *Thra1* located in the mitochondrial matrix (Wrutniak-Cabello et al., 2018). Thus, in MGs, T3 may mediate the light adaptation ability of retinal neurons by modulating the mitochondrial metabolism of local retinal cells via mtDNA transcriptional regulation.

Cones are exposed to intense sunlight and a wide range of light intensities and thus have relatively high energy requirements (Giarmarco et al., 2020). Therefore, MGs provide the critical molecule T3 to increase mitochondrial respiration to meet these energy demands. Combined with previous studies on retinal mitochondrial metabolism, we speculate that the changes in retinal mitochondrial genes detected at the single-cell level may stimulate cells to carry out aerobic respiration and provide ATP. This, in turn, allows cones to respond to and recover from light exposure rapidly, thereby increasing light responsiveness. In conclusion, our study revealed that activation of *Dio2* in MGs during light adaptation is involved in the regulation of mitochondrial metabolism and gene transcription in cones via the TH pathway and intercellular communication.

## Materials and methods

### Mouse husbandry

All animal procedures were approved by the Institutional Animal Care and Use Committees (IACUC) at the University of Science and Technology of China (USTC). Mice were maintained in standard housing facilities free of specific pathogens and were provided with free access to food and water. All mice used in the current study were over 2 months old. The mice were reared under 12-h/12-h light/dark normal circadian rhythm cycle conditions (200-lux white ambient illumination from 8:00 am to 8:00 pm), except for special experimental requirements. C57BL/6J mice were obtained from the Model Animal Research Center of Nanjing University. The *Gnat1*^−/−^ and *Gnat1*^−/−^; *Cnga3*^−/−^; *Opn4*^−/−^ mice were generously provided by K.-W. Yau from Johns Hopkins University. *Dio2*^−/−^ mice were generated using the CRISPR/Cas9 technique. Cas9 mRNA and sgRNAs were microinjected into fertilized embryos of C57BL/6J mice. An 8-bp deletion in exon 2 of the *Dio2* gene was confirmed using PCR with specific primers (forwards primer: 5ʹ- CAGCAGCAGTGTGTTAGTGTG-3ʹ, and reverse primer: 5ʹ- CTGGTGAGCTGCTGCACATC-3ʹ). Homozygous KO mice were born from a heterozygous intercross and used for phenotypic analyses in parallel with age- and sex-matched wild-type (WT) littermates as a control group. *Dio2*^*f*/*f*^ mice were obtained by inserting the loxp sequence before and after the CDS of *Dio2* exon 2, and special primers were used for identification (*Dio2*^*f*/*f*^ forwards primer 1: 5ʹ- TCTATAATGCTTGCTAGGCACTGAC-3ʹ, *Dio2*^*f*/*f*^ reverse primer 1: 5ʹ- TCTTCTCCGAGGCATAATTGTTACC-3ʹ, *Dio2*^*f*/*f*^ forwards primer 2: 5ʹ- GCTGACCGCATGGACAATAATG-3ʹ, and *Dio2*^*f*/*f*^ reverse primer 2: 5ʹ- CCAGTTGGGTTTGTTTCTGGTGA-3ʹ).

### Mouse retina preparation

Mice were kept under light and dark adaptation conditions: At 8:00 am (ZT0) on the day of the experiment, mice in both groups were exposed to light for 3 h under normal light conditions. At 11:00 am (ZT3), one group was transferred to a dark box for feeding in darkness (dark adaptation), while the other group was exposed to normal light conditions. At 2:00 pm (ZT6), retinal samples were obtained from both groups.

T3 *ex vivo* incubation: At 8:00 pm (ZT12) on the day before the experiment, mice were transferred to a dark box for feeding in darkness. From 10:00 am to 12:00 am on the day of the experiment, mice were treated in a dark room to obtain retinal samples. Samples from the two groups of mice were placed in control group solution (normal Ames’ solution: 120 mmol/L NaCl, 22.6 mmol/L NaHCO_3_, 3.1 mmol/L KCl, 0.5 mmol/L KH_2_PO_4_, 1.5 mmol/L CaCl_2_, 1.2 mmol/L Mg_2_SO_4_ and 6 mmol/L glucose, equilibrated with 5% CO_2_/95% O_2_) and experimental group solution (Ames’ solution + 100 nmol/L T3), with retinal samples then obtained after 1.5 h of incubation away from light.

### Single retinal cell dissociation

The retinal samples were transferred to 1 mL of digestive solution containing papain (1 mg/mL), collagenase (2 mg/mL), and DNase (200 μg/mL), slightly chopped, incubated and digested using a thermostatic oscillator (300 ×*g*, 37°C, 20–25 min) with a gentle pipette up and down every 5 min, and a microscopic examination was performed. Phosphate-buffered saline (PBS) containing 10% fetal bovine serum (FBS) was then added to end the digestion reaction. After low-speed centrifugation (200 ×*g*), the cells were suspended three times in 0.04% bovine serum albumin (BSA) and PBS and then filtered using a 40-μm cell strainer. Trypan Blue and 7-AAD staining were used for analysis and detection to ensure that the average survival rate of samples was greater than 90%. Single-cell cDNA libraries were prepared using a 10X Single Cell 3 v2 Reagent Kit according to the methods provided.

### scRNA-seq data preprocessing

Droplet-based raw data were processed using Cell Ranger (Version 2.1.1) (Stuart et al., 2019) against the mm10 mouse reference genome with default parameters. Single-cell read counts from all samples were first converted to individual Seurat objects using the Seurat (v3.2.2) analysis package in R (v3.6.3). First, the ambient RNA signal was removed using the default SoupX workflow (v1.5.0) (Young and Behjati, 2020). Meanwhile, to avoid the effects of doublets, we also removed potential doublets using DoubletFinder (McGinnis et al., 2019). To exclude poor-quality cells that may result from multiplets or other technical noise, we filtered cells that were considered outliers based on the number of expressed genes detected, sum of UMI counts and proportion of UMI counts for mitochondrial genes.

In the light-dark adaptation datasets, for quality control of each matrix, cells were filtered based on a minimum of 800 and maximum of 6,000 expressed genes per cell. Cells with mitochondrial gene percentages over 30% were discarded. For the five matrices, we used batch correction with Seurat v3.0 to sequentially define pairwise anchors across the five batches and integrate the data to remove possible batch effects. In brief, each sample was split into its own Seurat object, then 2,000 variable genes were selected with “vst”, anchors were found using FindIntegrationAnchors (k.anchor = 5, k.filter = 300, k.score = 30, dims = 1:50, max.features = 200, anchor.features = 3,000), and the data were integrated with IntegrateData (k.weight = 100, dims = 1:50). The genes were then removed if they were detected in fewer than three cells. This resulted in a dataset of 25,176 single cells, including 14,909 cells and 21,874 genes from Light, 10,267 cells and 21,874 genes from the dark. In the integrated object, counts were log-normalized for each cell using the natural logarithm of (1 + counts per 10,000) and scaled using ScaleData. Cells were visualized using two-dimensional *t*-distributed stochastic neighbor embedding (tSNE). Principal component analysis (PCA) was performed on the integrated data (npcs = 50), and 40 PCs were used in FindNeighbors. We used the FindClusters function with resolution = 0.2 in Seurat v3.2.2 and retained 17 clusters.

### T3 dataset downsampling

To eliminate the effect of cell number on the T3 dataset results, we randomly extracted 3,000 cells from two T3 incubation samples and reanalyzed them using the following parameters. For quality control of each matrix, cells were filtered based on a minimum of 200 and a maximum of 4,000 expressed genes per cell. For the control sample, cells with mitochondrial gene percentages over 15% were discarded. For T3_1, sample cells with mitochondrial gene percentages over 20% were discarded. For T3_2, sample cells with mitochondrial gene percentages over 25% were discarded. We used Seurat to remove the batch effect. For each sample, 4,000 variable genes were selected with “vst”, anchors were found using FindIntegrationAnchors (k.anchor = 5, k.filter = 300, k.score = 30, dims = 1:50, max.features = 200, anchor.features = 2,000), and the data were integrated with IntegrateData (k.weight = 100, dims = 1:50). This resulted in a dataset of 10,085 single cells, including 6,000 cells and 31,052 genes from the T3, 4,085 cells and 31,052 genes from the control. In the integrated object, counts were log-normalized for each cell using the natural logarithm of (1 + counts per 10,000) and scaled using ScaleData. Cells were visualized using a two-dimensional tSNE. PCA was performed on the integrated data (npcs = 50), and 20 PCs were used in FindNeighbors. We used the FindClusters function with resolution = 0.1 in Seurat v3.2.2 and retained 14 clusters.

### Detection of DEGs in each cluster

Cluster-specific marker genes for transcriptional subpopulations were identified using the FindAllMarkers Seurat function with the Wilcoxon rank-sum test. To remove contamination in each cell type obtained above, sub-clustering of major cell clusters was performed in the same workflow. Some groups are contaminated with rods and subsequently removed. Differential gene expression analysis between the control and dark/light adaptation was performed using the “FindMarkers” function with the “bimod” setting in the Seurat package. DEGs were filtered based on fold-change > 0.30 with natural log and *P*-value adjust < 0.05. In the T3 dataset, significant DEGs were filtered by fold change > 1 with natural log and *P*-value adjust < 0.05.

### Bulk RNA-seq analysis

FASTQ sequences were mapped to the mouse genome mm9, which was downloaded from the UCSC genome browser website and aligned using STAR (Dobin et al., 2013). After obtaining the expression matrix, differential expression analysis was conducted using the DESeq2 (Love et al., 2014) package for R. Differentially expressed genes were determined based on an adjusted *P* value using the Benjamini–Hochberg procedure to correct for multiple comparisons with a false discovery rate (FDR) < 0.05 and |log_2_ fold-change| > 1.5.

### Assessment of receptor-ligand interactions

CellChat (v1.0.0) (Jin et al., 2021) was used to evaluate cell‒cell interactions and significant pathways. To identify potential cell‒cell interactions perturbed or induced under light and dark adaptation, the differentially expressed ligands and receptors in rods, cones, MGs, RBs, CBs, GCs, HCs, AC, and MiGs were analyzed. We followed the official workflow, and normalized data were loaded into CellChat. After creating CellChat objects, we used CellChatDB.mouse and set the Cell‒Cell Contact as the database. In addition, to make the database more complete, neural transmitter related (Kertes et al., 2011) ligand receptors for Nuclear Hormone Receptor gene set and Hormone Biosynthetic Process gene set in published cell interaction literature (Ximerakis et al., 2019) were added to CellChatDB.mouse’s database to generate a new ligand‒receptor pair reference data. Then, parameters with identifyOverExpressedGenes (thresh.pc = 0.35) were used to identify putative interaction pairs, and the results were displayed as circos plots.

### Weighted correlation network analysis (WGCNA)

WGCNA analysis was performed using the relative average expression matrix of the retinal cell types (normalized gene expression values of the genes under light and dark adaptation) (Langfelder and Horvath, 2008). The genes included in the matrix were derived from the top marker genes of each cell type. After selecting these genes to form a new expression matrix, the matrix was normalized. We transformed this normalized matrix to a similarity matrix based on the pairwise Pearson correlation among genes and then converted the similarity matrix into an adjacency matrix. Using the dynamic hybrid cutting method, WGCNA identified six gene modules, each of which contained a set of genes that tended to be coexpressed in a certain cell type. The modules of interest were shown as heatmap. Gene lists in modules of interest were extracted and submitted to DAVID v6.7 (Huang da et al., 2009) for KEGG pathway analysis.

### Gene set enrichment analysis

The gene matrix [log_2_ fold-change (light/dark)] was arranged in descending order and then used the R package GSEABase to calculate the enrichment score and *P* value of the gene set downloaded from MSigDB (Liberzon et al., 2011) in the gene matrix. Here, *P*-value < 0.05 was considered as significantly enriched.

### RNA extraction and quantitative reverse transcription PCR (RT-qPCR)

Each sample contained two retinae from the same mouse. Total RNA was extracted using TRIzol reagent, and cDNA was obtained by reverse transcription (Takara). All primers used in this study are described in Table S3. qRT-PCR was performed using the Light-Cycler 96 system (Roche) following the manufacturer’s recommended protocols. *Gapdh* gene was used as the reference gene, and the comparative Ct method (2^−∆∆Ct^ method) was used to detect mRNA expression changes in different samples. For the detection of mRNA changes in different light and dark adaptation processes, samples were taken every 3 h. Finally, the relative expression level at the ZT0 time point was used as the standard to measure the fold-change of each ZT point.

### RNAscope assay

Retinal samples were obtained from the two groups of mice with different adaptation patterns, consistent with the conditions of light/dark-adapted single-cell sequencing samples. Eyeballs were collected at 2:00 pm (ZT6), placed in 4% paraformaldehyde (PFA) solution, fixed at room temperature for 2 h, and transferred to 30% sucrose in PBS overnight at 4°C. The cornea and lens were dissected and removed using a frozen microtome (Lecia) to obtain 16-μm retinal sections. The sections were postfixed with 4% PFA and then assayed using an RNAscope Multiplex Fluorescent Reagent Kit v2 (ACD) according to the manufacturer’s instructions. A Fluoview FV1200 microscope (Olympus) was used for image recording, and ImageJ was used for image analysis.

### Enzyme-linked immunosorbent assay (ELISA) for measurement of thyroid hormone

The mice were divided into two groups. At 8:00 pm (ZT12), the day before collecting samples, both groups were transferred to a dark box. On the day of the experiment, 1 h before collecting samples, one group was removed and placed under normal light feeding conditions, while the other group was kept in the dark box. Experimental samples were collected from both groups, with each sample containing four retinae. Cell lysis solution was added to each sample, which was then cleaved using an acryogrinding instrument. After high-speed centrifugation, the supernatant was obtained. Concentrations of T3 in the samples were analyzed using a Mouse/Rat T3 ELISA Kit (Calbiotech). All experimental procedures were followed the manufacturer’s instructions, and the absorption of each sample was determined at 450 nm (SpectraMax iD5). The standard curve was generated using four-parameter exponential nonlinear regression in PRISM, and the concentration of T3 in each sample was calculated. At the same time, the total protein content in the samples was detected to normalize the T3 content between samples.

### Analysis of mitochondrial oxygen consumption rate (OCR)

Before the experiment, the mice were dark-adapted overnight. Eyeballs were obtained from dark-adapted mice anaesthetized with isoflurane under dim red light. Fresh dissected retinal samples were incubated in Ames’ solution (120 mmol/L NaCl, 22.6 mmol/L NaHCO_3_, 3.1 mmol/L KCl, 0.5 mmol/L KH_2_PO_4_, 1.5 mmol/L CaCl_2_, 1.2 mmol/L Mg_2_SO_4_, and 6 mmol/L glucose) with oxygen for 1.5 h, with 100 nm T3 then added to the incubation solution of the experimental group. After incubation, three 1-mm biopsy punches were taken around the optic nerve of each retina using a 1-mm biopsy punch. The individual punches were transferred to a 24-well islet capture microplate. Each well contained 500 μL of intracellular fluid (135 mmol/L NaCl, 10 mmol/L HEPES, 3.1 mm KCl, 0.5 mm KH_2_PO_4_, 1.5 mm CaCl_2_, 1.2 mm MgSO_4_, 6 mmol/L glucose, pH adjusted to 7.4 by NaOH) bubbled with 95% O_2_/5% CO_2_. The OCR was measured using a Seahorse Extracellular Flux Analyzer XF24 (Agilent Technology) and Mito Stress Test Kit (Agilent Technology, 103015-100). After measuring the initial OCR, 20 μmol/L oligomycin was added to inhibit ATP production, followed by the addition of 1 μmol/L Carbonyl cyanide-4- (trifluoromethoxy) phenylhydrazone (FCCP), and finally by 1 μmol/L rotenone and 1 μmol/L antimycin A (R + A). Five cycle measurements were obtained after each treatment, and each data point was the average of 11 individual wells for each treatment. The results were calculated according to the protein level.

### *Ex vivo* ERG analysis

*Gnat1* ^−/−^ mice were dark-adapted for more than 3 h before *ex vivo* ERG recording. Eyeballs were obtained from dark-adapted mice anaesthetized with isoflurane under dim red light. After dissecting the eyeballs under red light using a microscope, the fresh dissected retinal samples were placed in Ames’ solution and incubated with oxygen for 1.5 h. Then, 100 nm T3 was added to the incubation solution of the experimental group. For ERG recordings of mouse retinae, several synaptic transmission blockers (STBS) were added to Ames’ solution to isolate the specific a-wave light response of photoreceptor cells [20 μmol/L l-(+)-2-amino-4-phosphonobutyric acid (L-AP4) to the solution to block on-bipolar cell signals, 20 μmol/L 6-cyano-7-nitroquinoxaline-2,3-dione (CNQX) to block AMPA/kainate receptors, and 20 μmol/L d-2-amino-5-phosphonovalerate (D-AP5) to block *N*-methyl-d-aspartate receptors]. The retinal samples were flat-mounted on the recording chamber (photoreceptor side up), and ERG recordings were conducted between a ground electrode in the bottom chamber and a recording electrode positioned above the retina. The retina was stimulated with 20-ms flashes at 520 nm, with an intensity gradient of 80.71–7.89 × 10^5^ photons/μm^2^ for each flash. The recorded signals were amplified, low-pass filtered at 20 Hz, and digitized for further analysis. The data were analyzed using a custom program in GraphPad Prism v5.0 and expressed as the mean ± standard error of the mean (s.e.m.)

### *In vivo* ERG analysis

*Dio2* ^*f*/*f*^ mice (6–8 weeks old) in the same litter were intravitreally injected with the AAV virus. The control group was injected with AAV-GFAP-GFP virus, and the experimental group was injected with AAV-GFAP-Cre-GFP virus. After injection, mice were kept in a normal rhythmic environment under 12:12-h light/dark cycles. ERG analysis was performed three weeks later. After overnight dark adaptation, the mice were given 300 lux of normal light for 1.5 h to allow rod cells to reach light saturation, and then pupils were dilated using 1% tropicamide. Before recording, the mice were anaesthetized using ketamine (40 mg/kg). To measure cone responses, rods were saturated with a steady-state 300 lux of normal light. Excitation light (495 nm) from the filter in front of a white LED was irradiated through the optical fiber to provide 20 ms flash stimulation, with an increased intensity gradient for each flash. The recorded signals were amplified, low-pass filtered at 20 Hz, and digitized for further analysis. The data were analyzed using a custom program in GraphPad Prism v5.0 and expressed as the mean ± s.e.m.

### Photocurrent recording of single rod cells in mouse retinae

Before rod suction pipette recordings, mice were dark-adapted overnight. Eyeballs were obtained from dark-adapted mice anaesthetized with isoflurane under dim red light. Before recording, the samples were incubated in solution for 1.5 h (with 100 nmol/L T3 added in the experimental group). The retinal samples were then flat-mounted on the recording chamber (photoreceptor side up). Imaging was recorded under infrared (650 nm) using an Olympus orthostatic microscope. Ames’ extracellular solution was used for recording. The outer segments of the rods were gently sucked into a glass pipetting tube (diameter 1.5–2.0 mm) filled with intracellular fluid. Excitation light (535 nm) from the filter in front of a white LED was irradiated through the imaging objective to provide 20-ms flash stimulation. The light intensity gradient was from 0.64 to 1.1 × 10^3^ photons/μm^2^. An Axon 700B amplifier and a Digital 1440A interface were used to perform 50-Hz low-pass filtering and 25-kHz sampling of the recorded data. The data were analyzed using a custom program in GraphPad Prism v5.0 and expressed as the mean ± s.e.m.

### Statistical analyses

All data are represented as the mean ± s.e.m.. Comparisons between two groups were made using *t*-tests. Two-way analysis of variance (ANOVA) was used for comparisons of more than two groups. Quantification graphs were analyzed using GraphPad Prism (GraphPad Software).

## Supplementary information

The online version contains supplementary material available at https://doi.org/10.1093/procel/pwad007.

pwad007_suppl_Supplementary_Figures

pwad007_suppl_Supplementary_Table_S1

pwad007_suppl_Supplementary_Table_S2

pwad007_suppl_Supplementary_Table_S3

pwad007_suppl_Supplementary_Table_S4

## Data Availability

The raw single-cell RNA-seq and bulk RNA-seq data used in this study have been deposited in the Genome Sequence Archive (GSA) in the National Genomics Data Center under accession number CRA006518.

## Abbreviations

AAV: adeno-associated virus
ACs: amacrine cells
ANOVA: analysis of variance
ATP: adenosine triphosphate
BCs: bipolar cells
BSA: bovine serum albumin
CB: cone bipolar cell
CNQX: 6-cyano-7-nitroquinoxaline-2,3-dione
DACs: dopaminergic amacrine cells
D-AP5: d-2-amino-5-phosphonovalerate
DEGs: differentially expressed genes
ELISA: enzyme-linked immunosorbent assay
ERG: electroretinography
FBS: fetal bovine serum
FCCP: carbonyl cyanide-4-(trifluoromethoxy) phenylhydrazone
FDR: false discovery rate
GCAP: guanylate cyclase activating protein
GCs: ganglion cells
GFP: green fluorescent protein
GSA: genome sequence archive
GSEA: gene set enrichment analysis
HCs: horizontal cells
HE: hematoxylin and eosin
ILM: inner limiting membrane
INL: inner nuclear layer
IPL: inner plexiform layer;
ipRGCs: intrinsically photosensitive retinal ganglion cells
IS: inner segment of photoreceptors
KEGG: Kyoto Encyclopedia of Genes and Genomes
L-AP4: l-(+)-2-amino-4-phosphonobutyric acid
L-cone: long-wave cone photoreceptor
M-cone: medium-wave cone photoreceptor
MGs: Müller glial cells
MiG: microglia cell
mRNA: messenger RNA
OCR: oxygen consumption rate
OLM: outer limiting membrane
ONL: outer nuclear layer
OPL: outer plexiform layer
OS: outer segment of photoreceptors
PBS: phosphate-buffered saline
PFA: Paraformaldehyde
qRT-PCR: quantitative real-time polymerase chain reaction
R + A: rotenone with antimycin A
RB: rod bipolar cell
S-cone: short-wave cone photoreceptor
scRNA-Seq: single cell RNA sequencing
STBS: synaptic transmission blockers
TFAM: transcription factor A
TH: thyroid hormone
TKO: Gnat1^−/−^; Cnga3^−/−^; Opn4^−/−^ triple knockout mice
TRs: thyroid hormone receptors
WGCNA: weighted correlation network analysis
ZT: Zeitgeber time

## Code availability

All codes for data analysis are included in Table S4.
