# Supplementary material for: Single-cell profiling reveals Müller glia coordinate retinal intercellular communication during light/dark adaptation via thyroid hormone signaling
Source: Protein Cell. 2023 Feb 21;14(8):603–17. doi: 10.1093/procel/pwad007 (PMC10392031; doi:10.1093/procel/pwad007)
Supplement: pwad007_suppl_Supplementary_Figures [file pwad007_suppl_supplementary_figures.pdf]

Supplementary Figure 1: Single-cell RNA-seq information of mouse retinal samples under light/dark adaptation

A-B: t-SNE plot of 25 176 single-cell transcriptomes (14 909 and 10267 from dark- and light-adapted samples, respectively). Each sample contained four retinae.

C: Number of genes and transcripts across clusters. Violin plot distributions of the number of genes (top) and transcripts (unique UMIs, bottom) detected in each cluster (n=5 samples). Cluster numbers as in Fig. S1A.

D: Heatmap of cluster analysis of 17 cell groups. Each group was clustered according to the top 15 genes with the highest expression in cells, P value adjusted < 0.05.

E: Expression patterns of known markers for different cell types in mouse retinae displayed in t-SNE plots (gray, no expression; red, relative expression).

Supplementary Figure 2: Quality control of scRNA-seq with *ex vivo* T3 incubation

(A) Dot plots show the expression of different TH transporter genes in each retinal cell type. The color of each dot indicates the average gene expression, and size indicates the percentage of cells in the cluster.

B: The average expression of *Slco1c1*, *Slco3a1*, *Slco5a1*, *Slc16a2*, *Slc7a5* and *Slc7a8* in different retinal cell types under light/dark adaptation.

C-D: t-SNE plot of 10 085 single-cell transcriptomes (4 085 from control and 6 000 from two T3 incubation samples). Each sample contained four retinae.

E: Number of genes and transcripts across clusters. Violin plot distribution of the number of genes (top) and transcripts (unique UMIs, bottom) detected in each cluster (n = 3 samples). Cluster numbers as in Fig. S3B.

F: Heatmap of cluster analysis of seven cell groups, with each group clustered according to the top 15 genes with the highest expression in cells, P-value adj < 0.05.

G: The expression patterns of known cell type markers in retinal samples displayed in t-SNE plots (gray, no expression; red, relative expression).

H: GSEA enrichment map of the respiratory electron transport pathway in cones and MGs under T3 incubation.

I: Experimental workflow for retinal energy metabolism test with T3 incubation *ex vivo*. Samples were placed in Seahorse XF24 Islet plates during energy metabolism testing.

Supplementary Figure 3: Dark-adapted ERG analysis in AAV-GFAP-Cre/GFP-injected *Dio2*$^{f/f}$ mice

(A) Schematic of single-cell rod suction pipette recording of rods from T3-incubated mouse retinae *ex vivo*.

B-C: Representative flash response from 0.64 to 1.1×10^3 photons•μm^{-2}. Tiny colored vertical bars on the x-axis indicate the time of light flashes.

D: Quantification of light intensity-response curves of rods from normal incubation (n = 8) and T3 incubation (n = 9) with light intensities from 0.64 to 1.1×10^3 photons•μm^{-2}. Intensity-response data are shown as mean ± s.e.m. intensity-response curves. n.s.>0.05, Two-way ANOVA.

E: Representative images of AAV infection in *Dio2*$^{f/f}$ mouse retinae with the injection of AAV-GFAP-Cre/GFP virus. Infected cells are marked in green, and all nuclei are marked in blue. Scale bar, 50 μm. Experiments were repeated three times independently with similar results.
F: Statistical analyses of the *Dio2* expression in GFAP-Cre and GFAP-GFP mice under light adaptation. Data are presented as the mean ± s.e.m., n = 9, *** $P < 0.001$.
G: Schematic diagram of the ERG recordings in AAV-GFAP-Cre/GFP virus-injected *Dio2*$^{f/f}$ mice under dark adaptation. The control group was injected with AAV-GFAP-GFP, and the experimental group was injected with AAV-GFAP-Cre-GFP. ERG recordings were performed three weeks after virus injection.
H: Statistical analyses of the *Dio2* expression in GFAP-Cre and GFAP-GFP mice under dark adaption. Data are presented as the mean ± s.e.m., n = 6 in AAV-GFAP-GFP injection group, n = 7 in AAV-GFAP-Cre-GFP injection group, *** $P < 0.001$.
I: Representative flash response images of *in vivo* ERG b-wave under different light intensities. Light with a 495 nm wavelength was used as the stimulus light, a 20 ms flash stimulus was given, and the light intensities ranged from 3.04×10^{-2} to 1.75×10^{4} photons•μm^{-2}.
J: Quantification of ERG b-wave amplitudes of light intensity-response curves with light intensities from 3.04×10^{-2} to 1.75×10^{4} photons•μm^{-2}. Intensity-response data are the mean ± s.e.m. intensity-response curves, the AAV-GFAP-GFP injection group n = 6, the AAV-GFAP-Cre-GFP injection group n = 7, n.s.>0.05, Two-way ANOVA.

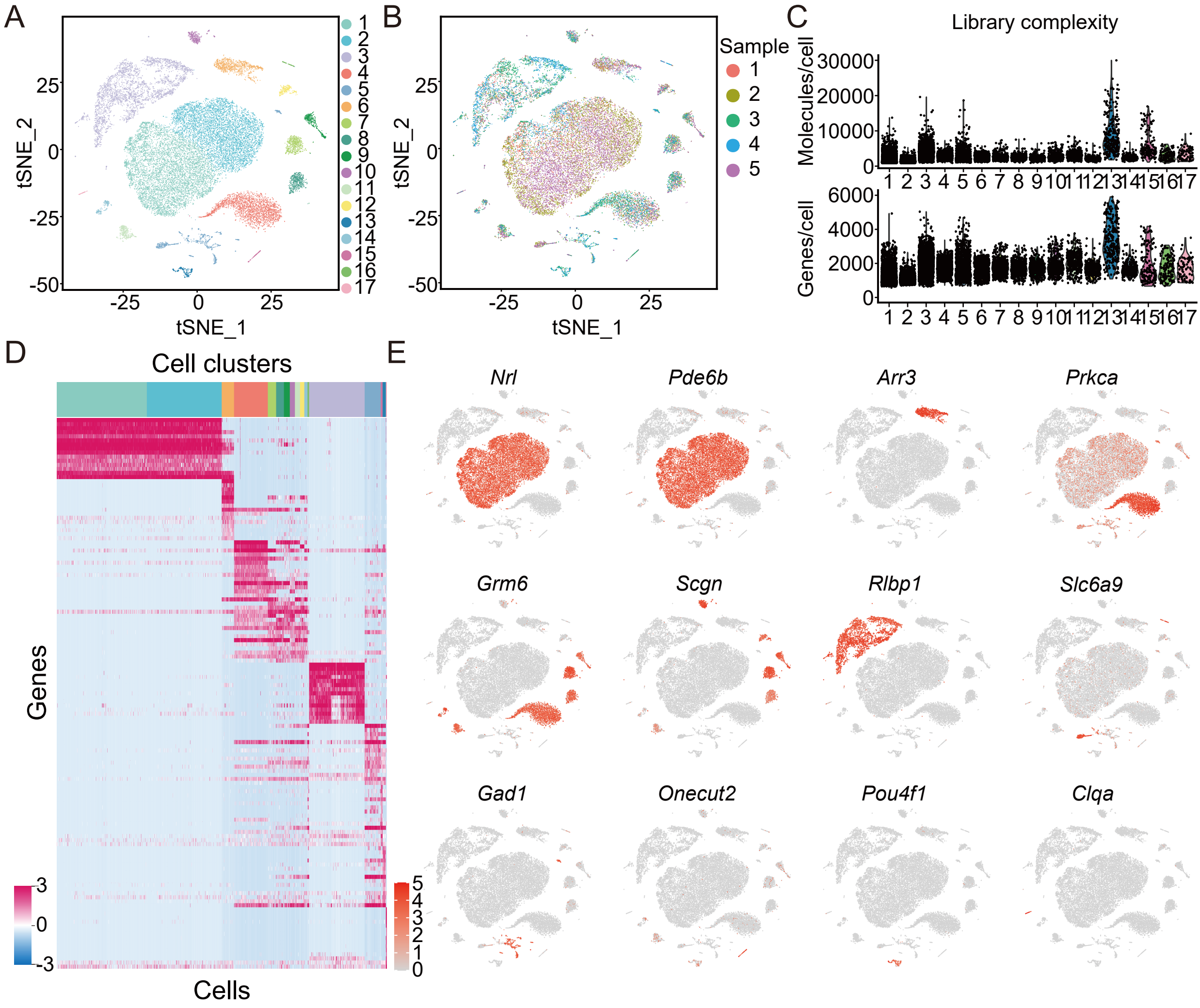

A
B
C
D
E
tSNE_1
tSNE_2
Sample
Library complexity
Molecules/cell
Genes/cell
Cell clusters
Genes
Cells
Nrl
Pde6b
Arr3
Prkca
Grm6
Scgn
Rlbp1
Slc6a9
Gad1
Onecut2
Pou4f1
Clqa

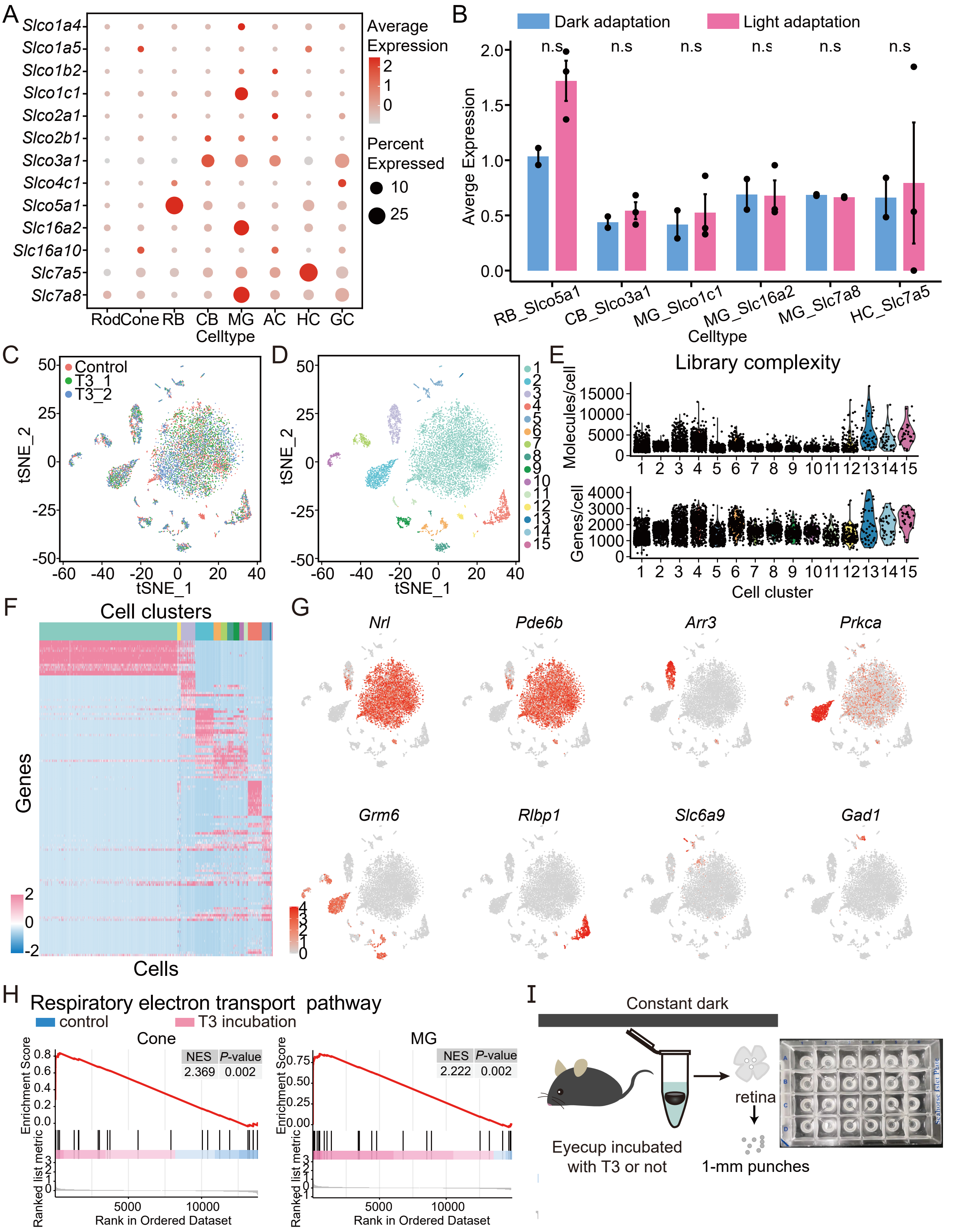

A
Slco1a4
Slco1a5
Slco1b2
Slco1c1
Slco2a1
Slco2b1
Slco3a1
Slco4c1
Slco5a1
Slc16a2
Slc16a10
Slc7a5
Slc7a8
Rod Cone RB CB MG AC HC GC
Celltype
Average Expression
Percent Expressed
B
Dark adaptation
Light adaptation
n.s
Averge Expression
RB_Slco5a1
CB_Slco3a1
MG_Slco1c1
MG_Slc16a2
MG_Slc7a8
HC_Slc7a5
Celltype
C
Control
T3_1
T3_2
tSNE_2
tSNE_1
D
E
Library complexity
Molecules/cell
Genes/cell
Cell cluster
F
Cell clusters
Genes
Cells
G
Nrl
Pde6b
Arr3
Prkca
Grm6
Rlbp1
Slc6a9
Gad1
H
Respiratory electron transport pathway
control
T3 incubation
Cone
MG
NES
P-value
2.369
0.002
2.222
0.002
Enrichment Score
Ranked list metric
Rank in Ordered Dataset
I
Constant dark
Eyecup incubated with T3 or not
retina
1-mm punches

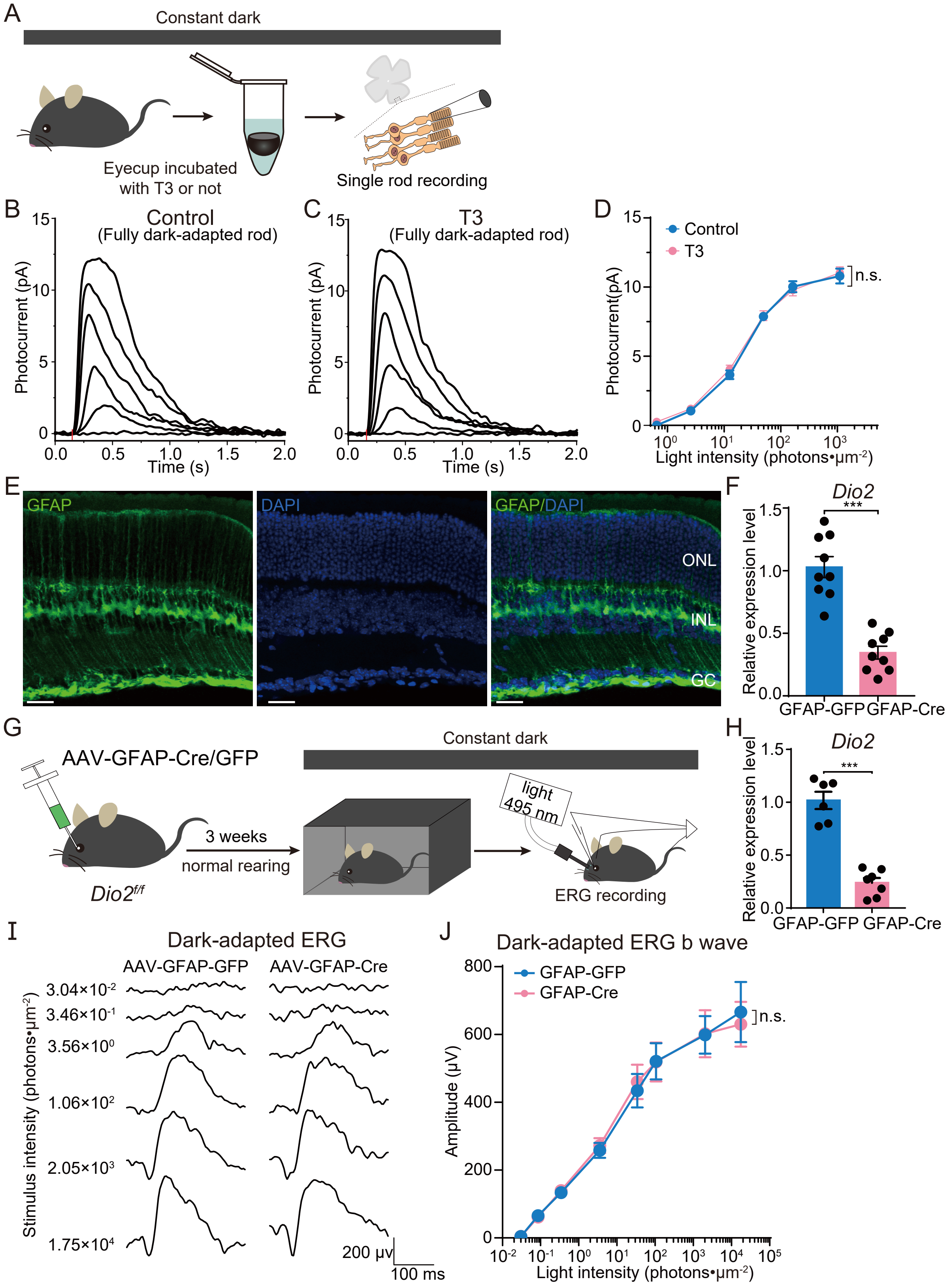

A
Constant dark
Eyecup incubated with T3 or not
Single rod recording
B
Control
(Fully dark-adapted rod)
Photocurrent (pA)
Time (s)
C
T3
(Fully dark-adapted rod)
Photocurrent (pA)
Time (s)
D
Control
T3
n.s.
Photocurrent(pA)
Light intensity (photons•μm-2)
E
GFAP
DAPI
GFAP/DAPI
ONL
INL
GC
F
Dio2
***
Relative expression level
GFAP-GFP
GFAP-Cre
G
AAV-GFAP-Cre/GFP
Constant dark
3 weeks
normal rearing
Dio2f/f
light
495 nm
ERG recording
H
Dio2
***
Relative expression level
GFAP-GFP
GFAP-Cre
I
Dark-adapted ERG
AAV-GFAP-GFP
AAV-GFAP-Cre
3.04×10-2
3.46×10-1
3.56×100
1.06×102
2.05×103
1.75×104
Stimulus intensity (photons•μm-2)
200 μv
100 ms
J
Dark-adapted ERG b wave
GFAP-GFP
GFAP-Cre
n.s.
Amplitude (μV)
Light intensity (photons•μm-2)
